# Identifying key performance tests in differentiating starting and substitute women Rugby players: a discriminant function analysis

**DOI:** 10.3389/fpubh.2025.1722936

**Published:** 2026-01-26

**Authors:** Cebrail Gençoğlu, Cemre Can Akkaya, Erkan Tortu, Abdulkadir Birol, Osman Ateş, Abdullah Demirli, İzzet İnce, Salih Çabuk, Süleyman Ulupınar, Serhat Özbay

**Affiliations:** 1 Faculty of Sport Sciences, Erzurum Technical University, Erzurum, Türkiye; 2 Faculty of Sport Sciences, Istanbul Yeni Yuzyil University, Istanbul, Türkiye; 3 Faculty of Sport Sciences, Trabzon University, Trabzon, Türkiye; 4 Faculty of Sport Sciences, Istanbul University-Cerrahpaşa, Istanbul, Türkiye; 5 Faculty of Sport Sciences, Ankara Yıldırım Beyazıt University, Ankara, Türkiye

**Keywords:** female athletes, female rugby players, performance testing, player selection, discriminant analysis

## Abstract

**Aim/background:**

This study aims to identify the performance tests with the highest discriminatory power for distinguishing between starting and substitute female rugby players.

**Method:**

A total of 24 female rugby players, who regularly participated in official league competitions and engaged in structured training on a regular basis, voluntarily participated in this study. The players were divided into two groups: starting players (age: 25.83 ± 3.81 years, height: 165.17 ± 5.83 cm, body mass: 61.08 ± 5.00 kg) and substitute players (age: 26.08 ± 3.12 years, height: 163.75 ± 5.33 cm, body mass: 60.42 ± 4.52 kg). Wingate, sprints (30 m), squat jump test, visual reaction and VO_2max_ performance tests were administered to the participants. Independent sample t-tests were used to analyze the statistical differences between player groups. Cohen’s d effect sizes are also calculated and classified according to the Hopkins. Subsequently, discriminant function analysis was applied to determine which tests had higher discriminatory power between starting and substitute rugby players.

**Results:**

Statistical analysis revealed significant differences between starting and substitute players in several performance variables, with starting players showing superior results in all performance tests, characterized by t-values ranging from 1.572 to 4.934, *p*-values < 0.001 to 0.130, and Cohen’s d effect sizes from 0.64 to 2.01. Discriminant function analysis identified the most discriminatory tests as sprint maximum sprint speed and Win_mean_ (both 87.5%), followed by 5 m sprint, Win_peak_, and performance decrease (PD) (all 83.3%).

**Conclusion:**

Maximum sprint speed and Wingate_mean_, with their superior discriminatory capabilities, are recommended for accurately assessing performance levels and differentiating player roles on the field, thereby enhancing training and selection processes in rugby.

## Introduction

Rugby is a high-intensity intermittent sport that includes repeated sprinting, tackling, and jumping actions. These high-intensity efforts are interspersed with short periods of low-intensity movements such as jogging or walking. The sport demands a blend of physical attributes, including speed, power, and endurance, to excel in different playing positions (1, 2). This complexity is further accentuated in rugby sevens, a variant that involves seven players per team and emphasizes rapid play and high endurance due to shorter match durations compared to the traditional 15-a-side rugby union (3). Numerous studies have aimed to establish the relationship between physical fitness attributes and match performance in both rugby union and rugby sevens. Research has consistently highlighted the significance of speed, power, and endurance in determining player effectiveness and overall team performance (3, 4). Specifically, sprint performance and anaerobic capacity are critical in high-intensity, intermittent sports such as rugby sevens (3, 5, 6).

The literature underscores the importance of tailored training programs based on specific performance indicators that correlate with successful outcomes in rugby. Performance indicators such as sprint speed, vertical jump height, and VO2max are crucial in differentiating player capabilities and their on-field roles (7–9). Accurate assessment of these attributes through reliable testing methods enables coaches to make informed decisions about player selection and development (10, 11). Understanding the physical and performance metrics that distinguish starting players from reserves has garnered research interest. Studies have highlighted the significance of anthropometric and fitness attributes in determining player performance and roles within a team. Factors such as sprint speed, aerobic capacity, and body composition are correlated with performance metrics in rugby, making them crucial for player selection and training (4). Given the physical demands of rugby and the critical role of performance indicators in differentiating player roles, this study aims to identify the key performance tests that most effectively distinguish between starting and reserve female rugby players. Discriminant function analysis, a statistical method used to predict group membership based on predictor variables, has been employed to identify key attributes that differentiate between groups of athletes. For example, Dobbin et al. (12) used discriminant function analysis to distinguish youth, academy, and senior rugby players based on their physical characteristics (12). This approach helps in understanding which tests have the highest predictive power in distinguishing starting players from reserves, thereby aiding in the optimization of training regimens and selection criteria (3, 11).

This study aims to provide valuable insights into the performance attributes that distinguish starting players from reserves in female rugby. By identifying the most discriminative performance tests, coaches can better assess and optimize player performance, thereby improving training and selection processes. The findings from this study will contribute to the growing body of knowledge on performance analysis in rugby and help enhance the competitive edge of female rugby players. It was hypothesized that starting players would outperform substitutes in all performance tests.

## Methods

### Participants

A total of 24 female rugby players, who regularly participated in official league competitions and engaged in structured training on a regular basis, voluntarily participated in this study. The players were divided into two groups based on their competitive season playing status: the starting players (*n* = 12) and the substitute players group (*n* = 12). All players were actively competing athletes in the national rugby league. Their weekly training regimen consisted of three structured sessions, including strength training, speed and conditioning drills, and technical–tactical rugby practice. Additionally, they participated in one official league match per week. The classification of players into these groups was determined according to their match participation frequency and coach-defined roles throughout the season. The starting players (age: 25.83 ± 3.81 years, height: 165.17 ± 5.83 cm, body mass: 61.08 ± 5.00 kg) and substitute players (age: 26.08 ± 3.12 years, height: 163.75 ± 5.33 cm, body mass: 60.42 ± 4.52 kg) regularly participated in weekly training sessions at the local rugby club facilities and played once a week. All participants were free from injury and provided written informed consent to participate in the study. Inclusion criteria included regular participation in the competitive season, consistent training attendance, and being injury-free for the past 6 months. Players with recent injuries, irregular training habits, or missing test sessions were excluded. Ethical approval was provided by the Erzurum Technical University Ethics Committee (Approval: Meeting No. 08, Decision No. 4, Date: 04.07.2024).

### Experimental design

Before the commencement of the main experimental trials, all participants visited the laboratory to become familiar with the testing procedures of the study. This familiarization session took place 48 h prior to the main testing sessions. Participants included elite athletes who reported to the laboratory having abstained from alcohol, caffeine, and strenuous exercise for 48 h prior to testing. The study employed a cross-sectional comparative design, in which all performance tests were administered across two testing sessions separated by a 24-h recovery period. Each session was designed to assess different aspects of the athletes’ performance and physiological capacity (Table 1).

**Table 1:** Descriptive characteristics of the rugby players.

Variables	Starting players	Substitute players	*p*	ES
Age (years)	25.83 *±* 3.81	26.08 *±* 3.12	0.862	0.07 (T)
Body mass (kg)	61.08 *±* 5.00	60.42 *±* 4.52	0.735	0.14 (T)
Height (cm)	165.17 *±* 5.83	163.75 *±* 5.33	0.541	0.25 (S)
BMI (kg/m^2^)	22.38 *±* 1.34	22.53 *±* 1.34	0.781	0.11 (T)
Body Fat (%)	27.40 *±* 2.00	27.65 *±* 2.77	0.807	0.10 (T)
Muscle mass (kg)	46.55 *±* 3.33	46.19 *±* 3.23	0.793	0.11 (T)

The values are presented as mean ± SD, ES: Effect Size (Cohen’s d), BMI: Body Mass Index, *Significant difference between the groups (starting – substitute players) (*p <* 0.05), T: Trivial Effect Size, S: Small Efect Size. *Significant difference between the groups, *p* < 0.05.

On the first day of testing, after basic anthropometric measurements were collected, participants engaged in a standardized warm-up. This warm-up was followed by a series of performance tests including the Wingate test, visual reaction time assessment, vertical jumps, and sprints. Each performance test was carefully monitored, and verbal encouragement was provided by both the performance staff of the governing body and the research team to ensure maximum effort from the participants. Adequate rest periods were provided between tests to ensure optimal performance. On the second day, following the same standardized warm-up protocol, participants performed the VO_2max_ test. Throughout both testing sessions, participants were allowed to consume up to 500 mL of water ad libitum. The room temperature was consistently maintained between 20 and 24 degrees Celsius to ensure a controlled environment for all tests (13).

The standardized conditions and protocols were adhered to strictly to ensure the reliability and validity of the results. By adhering to these procedures, the study aimed to accurately measure the performance and physiological responses of the athletes under controlled conditions.

### Sprint test

Sprint performance was assessed with electronic timing gates (Microgate, Italy) placed at 0, 5, 20 and 30 meters. These gates were spaced 200 cm apart and set at a height of 100 cm. Participants started each sprint from a two-point athletic stance, positioned 30 cm behind the starting line. Two maximal 30-meter sprints were recorded. The participants’ maximal sprint speed was calculated based on the highest speed reached between the 20 m and 30 m marks during the 30-meter sprint test. The Anaerobic speed reserve was calculated by subtracting the maximal aerobic speed from maximal sprint speed and expressed in km/h (14).

### Jump test

Each jump was performed three times on a portable jump mat (Optojump, Italy), with a 15-s recovery period between repetitions. The CMJ was conducted with hands on hips, beginning with a rapid countermovement to 90° of knee flexion followed by a vigorous extension into a maximal vertical jump (15). The SJ was executed similarly, but without the countermovement, requiring a 2-s pause before initiating the concentric phase. A 2-min rest interval was provided between different tasks, and the best attempt of each jump was used for subsequent analysis.

### Wingate test

Participants engaged in a standardized warm-up routine, which entailed five intervals of 30 s each at an intensity of 100 W. These intervals were split into 20 s at a cadence of 60 rpm and 10 s at a cadence of 110 rpm, performed on a bike ergometer (model 894E, Monark, Vansbro, Sweden). Following this warm-up, the participants undertook the 30-s Wingate test, targeting the lower body with a resistance load calibrated to 0.075 kg per kilogram of body weight (16). The Wingate test involved a singular, maximal effort over 30 s (17). The saddle height was tailored to each participant’s stature, ensuring a knee flexion angle between 5 and 10 degrees when the foot was in the lowest central position. Participants were instructed to pedal at maximum speed. Participants were verbally motivated during the test to ensure maximal effort. Performance metrics such as peak power (PP), minimum power (MinP), and mean power (MP) were recorded, representing the highest and lowest power outputs observed during the test. The fatigue index (FI) was computed by determining the difference between PP and MinP, dividing this value by PP, and multiplying the result by 100.

### Assessment of VO_2max_ and maximal cardiorespiratory parameters

A maximal incremental exercise test was conducted on a motor-driven treadmill (h/p Cosmos, Saturn, Germany) under laboratory conditions to assess at VO_2max_ and other maximal cardiorespiratory parameters. The test protocol began with participants walking for 2 min at a speed of 3 km/h. Subsequently, the treadmill speed increased by 1 km/h every 90 s until the participant reached volitional exhaustion. The treadmill grade was consistently set to 1% throughout the test. Respiratory gas exchange and heart rate were continuously monitored using an automated breath-by-breath metabolic system (Cosmed K_5_, Rome, Italy). This metabolic system was calibrated following the manufacturer’s guidelines. Raw data collected during the test were manually filtered and averaged at 5-s intervals. For the determination of VO_2max_, VO_2_ data were additionally averaged across 30-s time epochs. The highest VO2 response recorded during any 30-s epoch was designated as VO_2max_. To confirm the attainment of VO_2max_, specific criteria were applied: a plateau in VO_2_ and heart rate response despite increasing running speed (18). The speed at which VO_2max_ was achieved was defined as maximal aerobic speed (19). The V-slope approach provided by Beaver et al. was used to calculate the VT (20).

### Visual reaction

The Witty SEM diagnostic system utilized in this study comprised eight photocells aligned on a wall, each spaced 20 cm apart (21). Participants were instructed to react as swiftly as possible with their dominant hand to photocells that illuminated blue. Each test included 40 visual reaction tasks, with 5 reactions required for each photocell. Visual stimuli were presented immediately following each participant’s response. The final outcome was determined by the best total reaction time recorded over two trials.

### Statistical analysis

Statistical analyses were conducted using SPSS 27 (IBM SPSS Statistics for Windows, Version 27. Armonk, NY, USA). A significance threshold of *α* = 0.05 was utilized. Descriptive statistics were computed, and the findings are displayed as mean ± standard deviation. The Shapiro–Wilk test was employed to verify the normality of the data distribution.

An independent sample t-test was used to determine whether there were significant differences between the starting and substitute players. Cohen’s d formula (22) was calculated as post-hoc tests to determine the effect size. Results were classified according to Hopkins (23) [<0.2 = trivial (T); 0.20–0.59 = small (S); 0.60–1.19 = moderate (M); 1.20–1.99 = large (L); 2.00–3.99 = very large (VL); and ≥4 = nearly perfect (NP)]. Furthermore, a discriminant function analysis was employed to identify which tests and protocols most effectively differentiated between starting and substitute rugby players. The discriminant function analysis was applied individually to each set of test scores and protocols. The structural coefficient was utilized to pinpoint the variables that distinguished between starting and substitute players, with a coefficient above 0.30 deemed significant for interpreting the linear vector (24).

## Results

The analysis of various physical parameters revealed that there were no statistically significant differences in age (t = −0.176, *p* = 0.862, d = 0.07), body mass (t = 0.343, *p* = 0.735, d = 0.140), height (t = 0.621, *p* = 0.541, d = 0.254), BMI (t = −0.281, *p* = 0.781, d = −0.115), body fat (t = −0.247, *p* = 0.807, d = −0.101), and muscle mass (t = 0.266, *p* = 0.793, d = 0.109), as all parameters demonstrated trivial to small effect sizes.

As shown in Table 2, the statistical analysis results demonstrate significant variations across performance variables between starting and substitute rugby players. For sprint-related performances, larger effect sizes were observed with the maximum sprint speed (t = 4.934, *p <* 0.001, d = 2.01, Very Large effect size) and the 5 m sprint (t = −4.137, *p <* 0.001, d = −1.69, Large effect size). The 30 m sprint and anaerobic speed reserve also demonstrated large effect sizes (t = −3.314, *p* = 0.007, d = −1.35, Large effect size; t = 3.040, *p* = 0.006, d = 1.24, Large effect size), indicating notable differences in short-distance speed capabilities among the groups. Power-related measures such as Win.peak (t = 4.157, *p <* 0.001, d = 1.69, Large effect size) and Win.mean (t = 3.815, *p <* 0.001, d = 1.56, Large effect size) also presented large effect sizes, signifying significant differences in peak and mean power outputs.

**Table 2:** Comparisons of strength-power variables of the rugby players.

Variables	Starting players	Substitute players	*p*	ES(d)
5 m sprint _(sc)_	1.16 ± 0.05	1.31 ± 0.12	<0.001	−1.69 (L)
30 m sprint _(sc)_	4.66 ± 0.13	5.62 ± 1.00	0.007	−1.35 (L)
Win_peak(W.kg-1)_	12.31 ± 0.80	11.02 ± 0.71	<0.001	1.69 (L)
Win_mean(W.kg-1)_	7.74 ± 0.47	7.12 ± 0.32	<0.001	1.56 (L)
PD _(%)_	63.27 ± 6.28	75.15 ± 8.84	<0.001	−1.55 (L)
Visual Reaction _(sc)_	18.83 ± 1.53	21.46 ± 2.10	0.002	−1.43 (L)
CMJ _(cm)_	34.83 ± 4.40	31.98 ± 3.92	0.109	0.68 (M)
SJ _(cm)_	33.00 ± 4.05	28.79 ± 5.07	0.035	0.92 (M)
Aerobic Capacity _(test duration (sc))_	691.58 ± 59.48	651.92 ± 64.06	0.130	0.64 (M)
Aerobic Power _(VO2max (ml.min.kg-1))_	46.25 ± 4.66	42.15 ± 4.82	0.046	0.86 (M)
ATRS _(km/h)_	10.03 ± 0.74	9.45 ± 0.87	0.096	0.71 (M)
Aerobic Speed_max (km/h)_	12.77 ± 1.19	11.42 ± 1.71	0.035	0.92 (M)
Anaerobic Speed Reserve _(m/s)_	14.70 ± 1.44	13.13 ± 1.06	0.006	1.24 (L)
Sprint speed_max (km/h)_	27.47 ± 0.96	25.39 ± 1.09	<0.001	2.01 (VL)

The values are presented as mean ± SD, ES: Effect Size (Cohen’s d), CMJ: Counter Movement Jump, SJ: Squat Jump, Win_peak_: Wingate peak power, Win_mean_: Wingate mean power, PD: Performance decrease, T: Trivial ES, S: Small ES, M: Moderate ES, L: Large ES, VL: Very Large ES.

Visual reaction times (t = −3.518, *p* = 0.002, d = −1.43, Large effect size) and the percentage of power decline (PD%) from the Wingate test (t = −3.799, *p <* 0.001, d = −1.55, Large effect size) showed large differences, pointing to distinct capabilities in power utilization and reaction under pressure. Vertical jump tests, including CMJ (t = 1.671, *p* = 0.109, d = 0.68, Moderate effect size) and SJ (t = 2.248, *p* = 0.035, d = 0.92, Moderate effect size), exhibited moderate effect sizes, reflecting moderate but noteworthy differences in explosive leg power.

Furthermore, aerobic capacity tests, characterized by test duration (t = 1.572, *p* = 0.130, d = 0.64, Moderate effect size) and VO_2max_ (t = 2.113, *p* = 0.046, d = 0.86, Moderate effect size), showed moderate effect sizes, indicating moderate distinctions in endurance capabilities. The anaerobic threshold running speed (ATRS) (t = 1.739, *p* = 0.096, d = 0.71, Moderate effect size) and maximum aerobic speed (t = 2.246, *p* = 0.035, d = 0.92, Moderate effect size) also revealed moderate differences, highlighting varying endurance levels and sprint capacity across player types. These findings suggest that while explosive power and sprint speed are markedly different between starting and substitute players, the variations in jumping ability and aerobic capacity are less distinct but still relevant in distinguishing performance levels in competitive settings.

**Figure 1:**
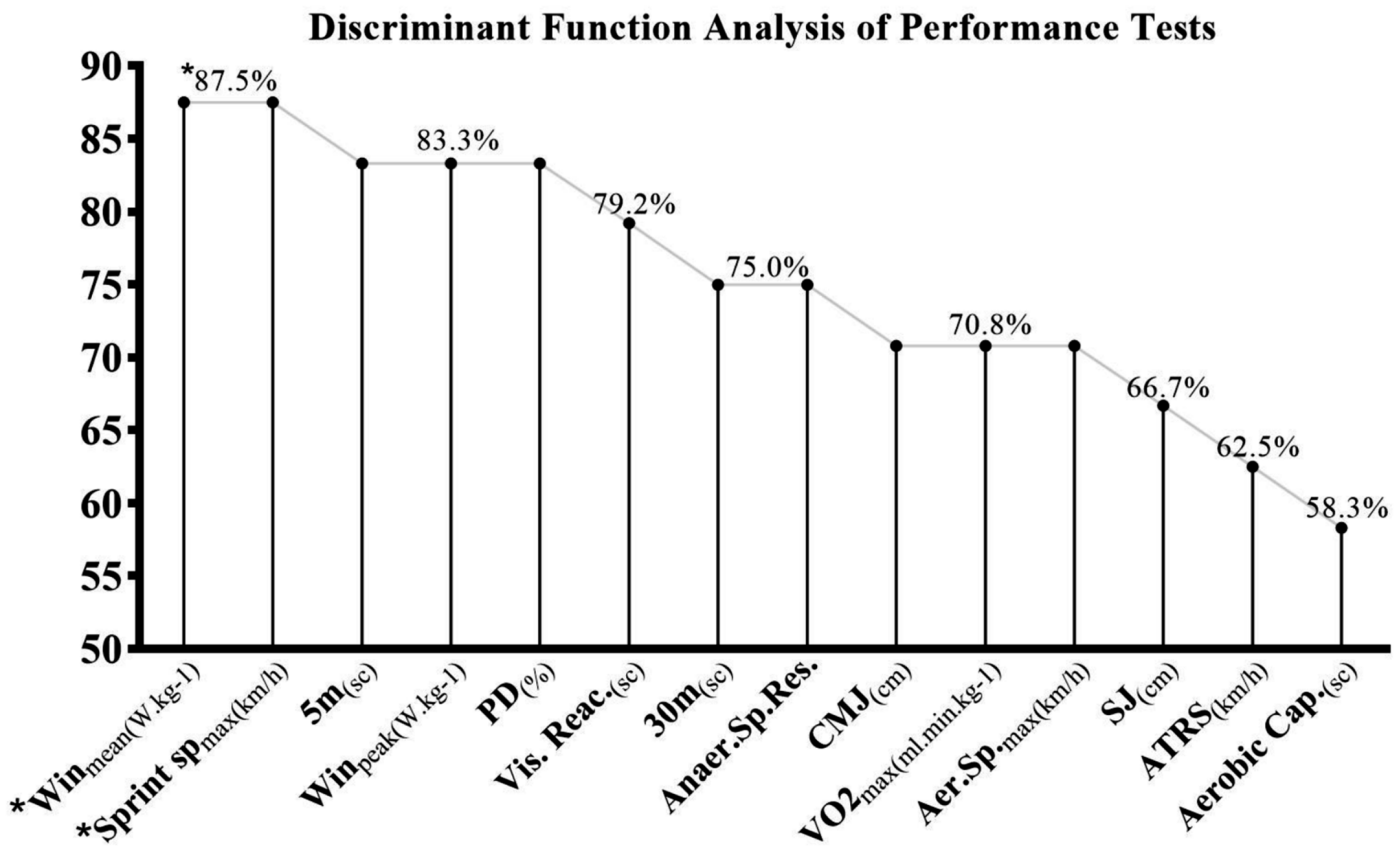
Discriminant function analysis results of the starting and substitute rugby players. Highlighting the highest discriminant scores among the various performance tests.

Figure 1 presents the results of the discriminant function analysis. According to these findings, the tests with the highest discriminatory power between starting and substitute rugby players were maximum sprint speed and wingate mean power, both with discrimination percentages of 87.5%, followed by the 5 m Sprint, Wingate Peak Power and Performance Decrease (PD) tests, each with a discrimination percentage of 83.3%. Conversely, tests such as the Squat Jump (SJ), Anaerobic Threshold Running Speed (ATRS), and Aerobic Capacity Test Duration exhibited lower discriminatory power, with percentages of 66.7, 62.5, and 58.3%, respectively.

## Discussion

This study aimed to identify performance tests with the highest discriminatory power for distinguishing between starting and substitute female rugby players. The results indicated significant differences between starting and substitute players in several performance variables, with starting players showing superior outcomes in performance parameters such as maximum sprint speed, Wingate mean power, and anaerobic speed reserve. These findings are consistent with previous research highlighting the importance of speed, power, and endurance in rugby performance (2, 3, 14, 25). Our results showed that starting players had significantly better sprint performance, particularly in the 5 m and 30 m sprints, compared to substitutes. This aligns with Ross et al. (9), who found that sprint speed over 10 m and 40 m had moderate to large negative correlations with key performance indicators such as line breaks and defenders beaten in rugby sevens (3), consistent with Twist et al. (2), who reported that movement demands and sprint capabilities are significant factors in player performance (2). Sprint speed is crucial in rugby for tasks such as making tackles, getting to the ball first, and scoring tries. The superior sprint performance of starting players suggests that acceleration and maximum speed are critical attributes for differentiating between starting and substitute players. These performance differences also reflect the demands of rugby match play, where rapid acceleration and short-distance sprinting are essential for winning ball contests, closing defensive gaps, and creating attacking opportunities. Likewise, higher anaerobic power supports repeated high-intensity actions such as tackling and ruck engagements, which are required more frequently from starting players. The work by Curtis et al. (26) on match-play demands and anthropometric characteristics of women’s rugby further supports the importance of physical attributes such as sprint speed and power in differentiating performance levels. Their review found that backs typically exhibit higher running demands and intensities, aligning with our findings that speed is a critical performance indicator (26).

The Wingate test results, particularly Wingate peak and mean power, were significantly higher in starting players. This indicates better anaerobic power and capacity, which are crucial for high-intensity efforts in rugby. This is consistent with findings from other studies that emphasize the role of lower body power in rugby, particularly for explosive movements like tackling and jumping (27). Sanford et al. (14) emphasized the importance of anaerobic speed reserve and its relationship with sport performance, further supporting our findings. The ability to sustain high power output is essential for starting players, who are often required to perform repeated high-intensity efforts during matches. The study by Moolman et al. (11) analyzed performance indicators that differentiate winning and losing teams in university-level rugby sevens. The study found that line breaks, tries scored, and successful tackles were key factors distinguishing winning teams (11). These findings support our study’s emphasis on the importance of sprint speed and anaerobic capacity, highlighting their critical roles in successful match outcomes.

The counter movement jump (CMJ) and squat jump (SJ) tests also reflected moderate to large differences, suggesting that starting players possess greater explosive leg power, which is essential for performance in high-intensity, intermittent sports such as rugby sevens (28). This is particularly pertinent in line-outs and defensive maneuvers, where vertical and horizontal power can significantly impact game outcomes (29, 30). Although differences in aerobic capacity (VO_2max_) were less pronounced than in anaerobic measures, starting players still demonstrated superior aerobic performance. The maximum aerobic speed and anaerobic threshold running speed also highlighted the superior aerobic and anaerobic endurance of starting players. This is in line with the findings of Ross et al. (9), who reported that higher aerobic capacity is associated with better match performance in rugby sevens, enabling players to maintain high work rates throughout the game (3). The ability to maintain high-intensity efforts with minimal decline is crucial in rugby sevens, where the game pace is relentless and recovery times are short (12, 30).

The substantial difference in visual reaction times between the two groups underscores the importance of cognitive and neuromuscular responsiveness in rugby. Faster reaction times enable players to respond more swiftly to dynamic game situations, enhancing their effectiveness on the field. This finding is in line with Gabbett et al. (1), who identified a strong correlation between visual-motor skills and overall game performance in rugby league. Owen et al. (31) emphasized the importance of various physical qualities such as body composition, muscular strength, power, and speed in rugby union players (31). Their review found that these qualities significantly differ by age and position, similar to the differentiation seen between starting and substitute players in our study. Brazier et al. (32) reviewed the anthropometric and physiological characteristics required for elite performance in rugby, noting that attributes such as speed, strength, and power are critical for success (32). These findings corroborate our results, where sprint speed and power measures were significant differentiators.

The integration of these findings into training and selection processes can greatly enhance team performance. Coaches should prioritize the development of sprint speed, anaerobic power, and reaction time in their training programs. Additionally, regular assessments using these key performance tests can help in making informed decisions about player roles and game strategies. By understanding and leveraging these performance metrics, teams can optimize their training regimens, ensuring that players are not only physically prepared but also capable of sustaining high performance throughout the game. This holistic approach to player development will likely lead to improved individual and team success on the field.

### Limitations

This study has some limitations. The small sample size (*n* = 24) and the inclusion of only female athletes from a single national league may limit the generalizability of the findings. Additionally, factors such as positional roles, recent training load, fatigue levels, and menstrual cycle phase were not controlled and may have influenced the results. Future studies with larger and more diverse samples are recommended.

## Conclusion

In conclusion, the current study identifies maximum sprint speed, Wingate mean power, and visual reaction time as key performance indicators that effectively distinguish starting players from substitutes in women’s rugby. These attributes should be prioritized in training and selection processes to enhance overall team performance. Future research should focus on integrating these performance tests into regular training assessments to further validate their predictive power in various rugby contexts.

## Data Availability

The original contributions presented in the study are included in the article/supplementary material, further inquiries can be directed to the corresponding author.
